# Rice Genomics: over the Past Two Decades and into the Future

**DOI:** 10.1016/j.gpb.2019.01.001

**Published:** 2019-02-13

**Authors:** Shuhui Song, Dongmei Tian, Zhang Zhang, Songnian Hu, Jun Yu

**Affiliations:** 1 BIG Data Center, Beijing Institute of Genomics, Chinese Academy of Sciences, Beijing 100101, China; 2 CAS Key Laboratory of Genome Sciences and Information, Beijing Institute of Genomics, Chinese Academy of Sciences, Beijing 100101, China; 3 University of Chinese Academy of Sciences, Beijing 100049, China

**Keywords:** Rice genome, Genomic diversity, Heterosis, Domestication

## Abstract

Domestic rice (*Oryza sativa* L.) is one of the most important cereal crops, feeding a large number of worldwide populations. Along with various high-throughput genome sequencing projects, rice genomics has been making great headway toward direct field applications of basic research advances in understanding the molecular mechanisms of agronomical traits and utilizing diverse germplasm resources. Here, we briefly review its achievements over the past two decades and present the potential for its bright future.

## Assembling and understanding the rice genomes using different sequencing approaches

Due to its limited genome size and diploidy, rice is an excellent choice among cereals for initiating genomic studies, serving as a model organism for plant biology and agricultural research. In 2002, the first two working draft genomes of the domestic rice (*Oryza sativa* L.) subspecies, *i.e.*, *japonica* (cultivar Nipponbare) and *indica* (cultivar 93-11), were successfully sequenced using whole-genome-shotgun (WGS) sequencing technology [1], [2]. The International Rice Genome Sequencing Project (IRGSP) Consortium was launched in September 1997, comprising research groups from Japan, the United States, France, South Korea, India, and China, aimed at delineating the Nipponbare genome using a map-based clone-by-clone (CBC) strategy [3], [4]. Meanwhile, there were two other efforts to sequence the same *japonica* cultivar from two private companies, Syngenta and Monsanto. Their sequencing data were publicly released in a controlled way and integrated into the IRGSP data. On the other hand, the *indica* genome project launched by the Chinese Superhybrid Rice Genome Project (CSRGP) went beyond genome sequencing and carried on with hybrid rice related studies [5]. In 2005, the complete genomes of both rice subspecies were released [6], [7], covering 95% of the 389-Mb genome. Furthermore, 37,544 non-transposable-element (nTE)-related protein-coding genes were predicted, with TEs accounting for 34.79% of the genomes. The finished genome is providing essential information for positional cloning and molecular studies.

High-quality genome assemblies offer unprecedented information and insights into genomics, evolution, and biology of rice, even when there were only two species with genome sequences available for comparative analysis – *Arabidopsis thaliana* and *O. sativa*. The first dilemma that needs to be resolved is where to place TEs, inside or outside a gene, as both plant genomes have considerable amount of TE-related contents. Distinct rules apply concerning how genes and genomes are organized differentially between angiosperms and vertebrates, whose collinearity has been appreciated by comparative genomicists. It appears that the two major lineages of the animal and plant kingdoms divergently made their distinct choices much earlier in evolution, perhaps since the birth of their unicellular ancestors [8]. However, it has not yet been solved which lower lineages share the same genomic parameters (such as intron size limits and ratio of genic *vs.* intergenic spaces) with the higher lineages within the kingdom of unicellular eukaryotes. Furthermore, the members of such a kingdom are yet to be firmly classified phylogenetically, and the decisive cellular mechanisms – RNA splicing machineries – are rather diversified, with some even cryptic among those organisms [9]. Insertions and dynamics of TEs are not only relevant to the history of related genes and their functional regulation within narrow taxonomic groups but also play significant roles in genome evolution among higher taxonomic groups, such as the ratio of the long terminal repeat (LTR) elements, *Gypsy vs. Copia* [10]. The second dilemma is related to the accelerated mutation mechanisms; in this case, transcript-centric positive GC gradients become obvious in the genomes of Gramineae [11], [12]. The gradients in GC content along the direction of transcription are not universal, which is shared by only the grass family of plants and warm-blooded vertebrates [11]. The third dilemma has to do with polyploidy and ancient whole genome duplication (WGD) events of plant genomes. Again, this dilemma is also shared between angiosperms and vertebrates, where polyploidy is prevalent among lower vertebrate and angiosperm lineages but abandoned by higher vertebrates, including reptiles, birds, and mammals [7], [13], [14].

One could certainly formulate more questions and dilemmas just at the genomic level. Moreover, it is well known that high-quality rice genome sequences and well-built references for its landraces, as well as subspecific and within-genus specific representatives, are essential for gene level comparative analysis. The revised reference genome assembly for Nipponbare (Os-Nipponbare-Reference-IRGSP-1.0) released in 2012 has been an excellent example, representing a product of concerted efforts using a variety of technologies, from optical mapping to CBC mapping, from clone-based assembly to heavy WGS coverage, and from long-read sequences of the Roche GS FLX to short-read sequences of the Illumina Genome Analyzer II platforms [15].

## Harnessing genome sequences to understand the biological basis of heterosis

Heterosis (hybrid vigor) is a phenomenon wherein F1 hybrids bear superiority for multiple agronomic traits attributable to the mix of genetic contributions of its parental inbred lines [16]. This is important in the use of F1 hybrid cultivars that are often elite crop varieties selected by breeders. To meet increasing food demands from population growth, scientists have cultivated hundreds of rice superhybrids in the past few decades. Remarkably, until 2018, a total of 131 rice cultivars had been officially approved as superhybrids with high-yield potential by the Ministry of Agriculture of China (http://www.ricedata.cn/variety/superice.htm). Among them, Liang-you-pei-jiu (LYP9) is one of the representatives developed using a two-line crossing between PA64S and 93-11. The featured high yield, fine grain quality, and strong biotic resistance (bacterial leaf blight and fungal blast diseases) of LYP9 are attributed to its intersubspecific heterosis [17]. In addition, another widely-planted hybrid Shan-you 63 (SY63), was generated using a method named the three-line hybrid system and bred from a cross between the male-sterile Zhen-shan 97A (ZS97A) and the restorer line Ming-hui 63 (MH63). SY63 features superior yield, multiple disease resistance, wide adaptability, and good eating quality, leading to large-scale plantation in southern and central China over the past three decades [18].

Over the past decades, valuable efforts have been devoted to understanding the biological basis of heterosis, including transcriptomic and epigenomic analyses [19], [20], [21]. Although several traditional models of heterosis (such as dominance, overdominance, and epistasis) have been suggested to explain the increased yield [22], we still do not understand the molecular mechanisms of heterosis. It is vital to have the high-quality genome sequences of the hybrid parents, which ultimately allows hybrid gene mapping free of sequence gaps and at a single-base resolution. Quality assembly of the parental genomes of hybrids (SY63 and LYP9) have been recently reported [23], [24], [25]. A map-based sequencing effort to assemble the parental genomes of SY63, *i.e.*, ZS97 and MH63, yielded 237 contigs for ZS97 and 181 contigs for MH63, covering 90.6% and 93.2% of their estimated genome sizes, respectively [24]. Similarly, with the support of CSRGP, the two parental genomes of LYP9, 93-11 and PA64S, have also been sequenced with high coverage [25]. Consequently, a series of variety-specific genes have been determined through comparative genome studies.

*Oryza glaberrima* is a domestic rice species in Africa that is reproductively isolated from Asian rice. In the early 2000s, aimed to generate ‘New Rice for Africa’ (NERICA), introgressions were carried out by crossing *O. sativa* and *O. glaberrima* cultivars, followed by recurrent back-crossing with the Asian rice parent. In 2017, the genomes of TOG5681 and CG14, parents of two NERICA generations, were sequenced [26]. The complete genome sequences would provide a rich resource, helping to tackle the issue of reproductive isolation for potential hybrid breeding from other distantly-related rice species.

To further reveal genetic elements of heterosis, low-coverage resequencing efforts have been reported in different superhybrid populations. One such effort dissected the immortalized second filial (IMF2) populations derived from the SY63 hybrid, showing the varied contribution of genetic components to yield traits [18]. For instance, overdominance/pseudo-overdominance contributes to a variety of yield-related traits (*e.g.*, the vigor of the yield, number of grains per panicle, and grain weight). In particular, the dominance × dominance interaction is closely associated with tillers per plant and grain weight. To map the heterotic loci at a fine scale, diverse superhybrid varieties and their inbred parental lines were massively resequenced [27], [28]. These include the recombinant inbred lines (RILs) of the super hybrid rice LYP9 [29] and the F_2_ lines from 17 representative hybrid rice crosses [30], [31]. The genetic architecture of yield traits and numerous superior alleles that contribute to heterosis were then proposed [27], [28]. Collectively, the availability of these parental genome sequences and hybrid population data provide rich resources for deciphering the genetic basis and molecular mechanisms of rice heterosis.

## Revealing rice domestication processes by exploiting additional genome sequences of *Oryza* species

**Figure 1:**
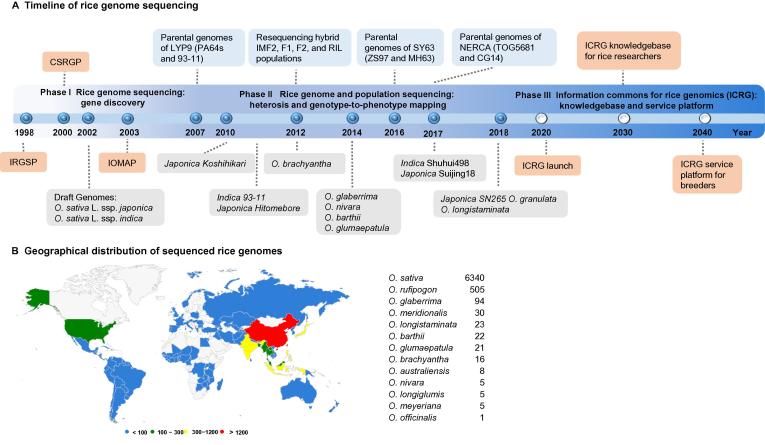
**The timeline of rice genomics and geographical distribution of sequenced rice genomes** **A.** Timeline of rice genomics. The solid circles indicate past events of rice genomics, including Phase I for rice genome sequencing and Phase II for rice genome and population sequencing. The open circles indicate projected future events for Phase III. In Phase III, the Information Commons for Rice Genomics (ICRG) is expected to be built around the year 2020, covering all rice genome assemblies of high quality and free of sequence gaps, becomes an open-access knowledgebase for all rice researchers around the year 2030, and becomes a service platform for rice breeders to design their new crops around the year 2040. **B.** Geographical distribution of sequenced rice genomes. The numbers of the genomes sequenced per country were color coded (blue, <100; green, 100–300; yellow, 300–1200; red, >1200), and the total numbers of sequenced genomes from different rice species are listed on the right. IRGSP, International Rice Genome Sequencing Project; CSRGP, Chinese Superhybrid Rice Genome Project; IOMAP, International *Oryza* Map Alignment Project; IMF2, immortalized second filial; RIL, recombinant inbred lines; NERICA, New Rice for Africa; ICRG, Information Commons for Rice Genomics.

More genome sequences of different *Oryza* cultivars have been constantly added to the rice knowledge base, including several strains of Asian cultivars and African cultivars (*O. glaberrima*) with the AA genome in the past few years (Figure 1A, Table 1). The deeply sequenced *O. sativa* genomes include IR64 (a conventional *indica* rice variety in China) [32], IR8 (also known as Miracle rice) [33], Swarna (an *indica* rice cultivar variety with low glycemic index) [34], Shu-hui498 (R498, an *indica* rice variety cultivar used as a restorer line in a three-line hybrid system) [35], DJ123, and N22 (*indica* rice with important disease resistance and abiotic tolerance alleles) [32], [33]. The African cultivars (*O. glaberrima*) provide an excellent resource for varietal improvement of *O. sativa*, as they harbor multiple important agronomic traits, especially for biotic and abiotic resistance. In 2014, CG14 was sequenced and assembled into 12 pseudomolecules with a total size of 316 Mb, and 33,163 gene models were annotated [26], [36]. Most of these cultivars were *de novo* sequenced with high coverage and often through integration of both short and long reads from the NGS platforms, which ensure high sequence coverage and moderate contiguity. For instance, comparative analyses of mutations in three orthologous genes *O. sativa Shattering 1* (*OsSh1*), *O. sativa Shattering 4* (*OsSh4*), and *qSh1* (LOC_Os01g62920) from African and Asian rice confirm independent domestication of genes controlling panicle shattering. However, to better identify and compare the orthologous loci, higher contiguity and quality rice genomes are still highly desired for future sequencing.

**Table 1:** **Genome sequence resources of *Oryza* species**

**Rice**	**Genome type**	**Genome size (Mb)**	**Sequencing depth (×)**	**Assembly level**	**Database**	**Weblink**	**PMID**
*O. sativa* L. ssp *japonica* (cv. Nipponbare) IRGSP1.0	AA	374.4	NA	Chr	RAP-DB	https://rapdb.dna.affrc.go.jp/	23299411 18089549
	AA	374.4	NA	Chr	RGAP	http://rice.plantbiology.msu.edu/	24280374
	AA	374.4	NA	Chr	NCBI, Ensembl	https://www.ncbi.nlm.nih.gov/assembly/GCF_001433935.1	24280374
*O. sativa* L. ssp. *japonica* (cv. Nipponbare)	AA	391.1	NA	Chr	NCBI	https://www.ncbi.nlm.nih.gov/assembly/GCA_000149285.1	15685292
*O. sativa* L. ssp. *japonica* (cv. Nipponbare)	AA	379.6	117	Chr	NCBI	https://www.ncbi.nlm.nih.gov/assembly/GCA_003865235.1	30448535
*O. sativa* L. ssp. *japonica* (cv. Nipponbare)	AA	355.6	110	Scaffold	CSHL	http://schatzlab.cshl.edu/data/rice	25468217
*O. sativa* L. ssp. *indica* (cv. 93-11)	AA	395.4	116	Chr	NCBI	https://www.ncbi.nlm.nih.gov/assembly/GCA_003865215.1	30448535
*O. sativa* L. ssp. *indica* (cv. 93-11)	AA	426.3	NA	Chr	NCBI, Ensembl	https://www.ncbi.nlm.nih.gov/assembly/GCA_000004655.2	11935017
*O. sativa* L. ssp. *indica* (cv. HR-12)	AA	389.8	93	Chr	NCBI	https://www.ncbi.nlm.nih.gov/assembly/GCA_000725085.2	26984283
*O. sativa* L. ssp. *indica* (cv. RP Bio-226)	AA	352.1	20	Chr	NCBI	https://www.ncbi.nlm.nih.gov/assembly/GCA_001305255.1	NA
*O. sativa* L. ssp. *indica* (cv. Shuhui498)	AA	391.0	120	Chr	NCBI	https://www.ncbi.nlm.nih.gov/assembly/GCA_002151415.1	28469237
*O. sativa* L. ssp. *indica* (cv. Minghui 63)	AA	398.8	228	Chr	NCBI	https://www.ncbi.nlm.nih.gov/assembly/GCA_001618785.1	27535938 27622467
	AA	387.4	120	Chr	NCBI	https://www.ncbi.nlm.nih.gov/assembly/GCA_001623365.2	
*O. sativa* L. ssp. *indica* (cv. Zhenshan 97)	AA	386.5	253	Chr	NCBI	https://www.ncbi.nlm.nih.gov/assembly/GCA_001618795.1	27535938 27622467
	AA	387.3	120	Chr	NCBI	https://www.ncbi.nlm.nih.gov/assembly/GCA_001623345.2	
*O. sativa* L. ssp. *indica* (cv. IR8)	AA	389.1	70	Chr	NCBI	https://www.ncbi.nlm.nih.gov/assembly/GCA_001889745.1	29358651
*O. sativa* L. ssp. *indica* (cv. IR64)	AA	345.2	110	Scaffold	CSHL	http://schatzlab.cshl.edu/data/rice	25468217
*O. sativa* L. ssp. *indica* (cv. Swarna)	AA	NA	10	NA	NA	NA	26068787
*O. sativa* L. ssp. *japonica* (cv. HEG4)	AA	342.0	220	Chr	NCBI	https://www.ncbi.nlm.nih.gov/assembly/GCA_000817615.1	29158416
*O. sativa* L. ssp. *japonica* (cv. Hitomebore)	AA	382.6	179	Chr	NCBI	https://www.ncbi.nlm.nih.gov/assembly/GCA_000321445.1	NA
*O. sativa* L. ssp. *japonica* (cv. A123)	AA	337.7	250	Chr	NCBI	https://www.ncbi.nlm.nih.gov/assembly/GCA_000817635.1	29158416
*O. sativa* L. ssp. *japonica* (cv. Koshihikari)	AA	382.2	15.7	Chr	NCBI	https://www.ncbi.nlm.nih.gov/assembly/GCA_000164945.1	20423466
*O. sativa* L. ssp. *indica* (cv. N 22)	AA	362.3	65	Chr	NCBI	https://www.ncbi.nlm.nih.gov/assembly/GCA_001952365.1	29358651
*O. sativa* L. ssp. *indica* (cv. DJ123)	AA	345.9	110	Scaffold	CSHL	http://schatzlab.cshl.edu/data/rice	25468217
*O. rufipogon* (cv.W1943)	AA	339.2	130	Scaffold	NCBI, Ensembl	https://www.ncbi.nlm.nih.gov/assembly/GCA_000817225.1	29358651
*O. barthii*	AA	308.3	110	Chr	NCBI, Ensembl	https://www.ncbi.nlm.nih.gov/assembly/GCA_000182155.2	29358651
*O. glaberrima* (CG14)	AA	303.3	30	Scaffold	NCBI, Ensembl	https://www.ncbi.nlm.nih.gov/assembly/GCA_000147395.2	29358651 25368197
*O. glumaepatula*	AA	372.9	135	Chr	NCBI, Ensembl	https://www.ncbi.nlm.nih.gov/assembly/GCA_000576495.1	29358651
*O. meridionalis*	AA	335.7	166	Chr	NCBI, Ensembl	https://www.ncbi.nlm.nih.gov/assembly/GCA_000338895.2	29358651
*O. punctata*	BB	393.8	130	Chr	NCBI, Ensembl	https://www.ncbi.nlm.nih.gov/assembly/GCA_000573905.1	29358651
*O. brachyantha*	FF	259.9	104	Chr	NCBI, Ensembl	https://www.ncbi.nlm.nih.gov/assembly/GCA_000231095.2	29358651
*O. nivara*	AA	338.0	102	Chr	NCBI, Ensembl	https://www.ncbi.nlm.nih.gov/assembly/GCA_000576065.1	29358651
*O. longistaminata*	AA	326.4	52.5	Scaffold	NCBI, Ensembl	https://www.ncbi.nlm.nih.gov/assembly/GCA_000789195.1/	NA
*O. granulate*	GG	777.0	117	Contig	GWH	http://bigd.big.ac.cn/gwh/Assembly/116/show	30271965

*Note*: ssp, subspecies; cv, cultivar; Chr, chromosome; NA, not applicable; RAP-DB, Rice Annotation Project database; RGAP, Rice Genome Annotation Project; GWH, Genome Warehouse.

Wild rice, being adapted to diverse geographical environments and exhibiting tolerance to biotic and abiotic stresses, can be exploited as important genetic resources and gene pools for molecular breeding. To date, there are 22 wild species in the genus *Oryza* that are distributed throughout the world, which are classified into ten genome types (AA, BB, CC, BBCC, CCDD, EE, FF, GG, HHJJ, and HHKK). Strategies to harness beneficial traits for crop improvement have been exemplified by the introgression of bacterial blight resistance gene *Xa21* from the wild rice *Oryza longistaminata* [37]. In 2003, the International *Oryza* Map Alignment Project (IOMAP) was initiated, with the aim of providing high-quality wild rice genomic resources for the discovery and utilization of beneficial genes and traits. So far, around 10 new reference genomes have been generated for wild rice species, including *O. nivara* (AA) and *Oryza rufipogon* (AA) from Asia; *Oryza barthii* (AA), *O. longistaminata* (AA), and *Oryza brachyantha* (FF) from Africa [36], [38]; *Oryza glumaepatula* (AA) from South America; *Oryza meridionalis* (AA) from Australia; as well as *Oryza punctata* (BB) from Africa and *Oryza granulata* (GG) from China [33], [36], [39]. In addition, two novel perennial wild rice species from tropical Australia with AA genomes were also sequenced (one is similar to *O. rufipogon* in plant morphology, and the other is similar to the annual *O. meridionalis*) [40]. The available genomes of wild progenitors and close relatives provide valuable resources for the identification of candidate genes and chromosomal regions selected during domestication [41]. To date, many genes with significantly lower diversity unique to cultivated rice have been identified, representing candidate regions for selective sweeps during domestication [42], [43], [44]. Comparative genomic analyses between the wild and the cultivated rice species are essential for mechanistic investigation of genome organization, gene family expansion, segmental duplication, *etc*.

## Dissecting genetic components for complex agronomic traits using genome-wide association studies

Building a comprehensive collection of landraces in terms of morphology, genetic diversity, and geography is fundamental for following genetic studies, such as genome-wide association studies (GWAS) of genotype-to-phenotype relatedness. Totally 773,948 rice accessions are available in various gene banks worldwide, with high genetic diversity [45]. For instance, there are ∼101,000 from the International Rice Genebank Collection (IRGC) at the International Rice Research Institute (IRRI), 61,470 from the China National Crop Gene Bank (CCGB) [46], and ∼18,000 from the United States Department of Agriculture (USDA) Rice Genebank [47]. Such collections enable population-based genome-wide studies for a broad scope of genetic and biological purposes.

An excellent example that utilizes a large number of rice accessions for GWAS by taking advantage of low-cost sequencing [48], [49] was shown by Han and his coworkers. They performed GWAS analyses and identified hundreds of known and new loci associated with 14 agronomic traits, covering two morphological characteristics (leaf angle and tiller number), four grain-related traits (grain width, grain length, grain weight, and spikelet number), three grain quality traits (gelatinization temperature, protein content, and amylose content), three coloration traits (apiculus color, pericarp color, and hull color), and physiological features (heading date, drought tolerance, and degree of seed shattering). Another comprehensive study that involved metabolic profiling and metabolic GWAS (mGWAS) identified hundreds of common variants that exert important influences on the production of secondary metabolites, accordingly revealing the biochemical relevance of gene–metabolite associations in rice [50]. Using the same sequenced materials, many genetic loci were revealed to be related to biochemical traits (*e.g.*, absolute content of chlorophyll), physiological features (*e.g.*, seed germination and degree of seed shattering), and content of mineral elements [51], [52], [53], [54], [55]. A further study reported a mapping effort for major-effect loci at the level of respectively causal SNPs, amylose content, seed length, and pericarp color by combining the diversity of the rice collection with low-coverage sequencing [56]. In short, such methods, by combining low-coverage genome-wide NGS-sequencing and GWAS, represent a complementary strategy for dissecting complex traits. However, there are still many genetic characteristics of important agronomic traits that have not yet been characterized. More studies are certainly required to reveal the genetic mechanisms by combining more phenotypic and genotypic data in natural populations in the near future.

## Analyzing genomic diversity through comparative genomic studies and data integration

More rice genomes with high quality sequences have provided unlimited opportunities for identifying genetic and other molecular markers, *e.g.*, SNPs, or simple sequence repeats (SSRs), which greatly facilitates population-based studies and marker-assisted breeding. To build an open-access information commons for rice genomics (ICRG) would be desirable to host genome assemblies and genome variations in the future and hopefully to integrate other large-scale genome annotations, including information from other omics-level collections. There are several databases available to be integrated, such as Ensembl Genome [57], Gramene [58], RAP-DB [59], [60], RGAP [15], dbSNP at NCBI [61], HapRice [62], SNP-Seek [63], [64], IC4R [65] in BIG Data Center [66], RiceBase [67], and RiceVarMap [68]. Notably, since 2017, the largest collection of rice SNPs we have organized is deposited in the GVM database (http://bigd.big.ac.cn/gvm/) by collecting and systemically analyzing sequence data of 5152 rice accessions (Figure 1B), in which a total of 18,616,579 SNPs and 9122 manually curated genotype-to-phenotype entries were integrated [69]. Furthermore, more than 10,000 novel, full-length, protein-coding genes and a high number of presence-absence variations (PAVs) were identified by resequencing a core collection of over 3000 Asian cultivated rice accessions from 89 countries, representing another component of species genetic diversity [70], [71], [72]. These sequence variations and resources are useful for population structure and diversity analysis. One such example is utilizing a large number of genomic variations for population-based phylogenomic analyses, providing evidence for the variety of the *O. sativa* gene pool in 5 major different groups – *indica, aus/boro, basmati/sadri, tropical japonica, and temperate japonica*, and in some unknown subpopulations related to geographic location [70].

SSR markers are another class of molecular marker that are widely used in gene mapping and breeding practice. They are also the primary choice for genotyping due to their high density, codominant inheritance, high allelic diversity, and highly reproducible methodology for detection. An excellent example for marker-assisted backcrossing breeding using SSR markers is to integrate rice blast resistance genes into a number of popular rice varieties to improve the blast disease resistance [73], [74]. The current genome coverage by both SSR and SNP markers is abundant for marker-assisted selection (MAS) and QTL (quantitative trait loci) mapping.

## Furthering function-centric and trait-centric gene cloning

The advances in rice genome sequencing projects have greatly boosted functional genomic studies. These studies aimed at exploring genes and regulatory networks of agronomically important traits and their application in varietal improvement, which include but are not limited to, yield, grain quality, reproductive development, and resistance to disease, pests, or abiotic stress. Over the past decades, scientists have used various platforms successfully for functional genomics, such as large-scale mutant libraries [75], [76], core germplasm collections, high-density gene expression arrays, and transcriptome sequencing [77]. In doing so, they have defined a number of trait-related genes with agronomic importance. Collectively, a total of 2358 rice functional genes (http://www.ricedata.cn/) were successfully cloned using map-based cloning techniques, including genes related to grain yield, grain size/weight, and grain quality [76], [78]. For instance, *GW5*, which regulates cell division during seed development, affects grain width [79], [80]; whereas the recently identified plant-specific transcription factor 13 (*OsSPL13*) appears to increase grain length [81].

Another typical example is genes involved in regulating plant architecture by controlling tillering and promoting panicle branching. For instance, *OsSPL14* (also known as ideal plant architecture, *1IPA1*), one of the *OsmiR156* targets that interacts with TEOSINTE BRANCHED1, negatively regulates tiller bud outgrowth [82], [83], [84]. In addition, *OsSPL14* also regulates the length and grain numbers of panicles by directly interfering with dense and erect panicle 1 (*DEP1*), a key protein determining panicle architecture [84]. The introduction of the *OsSPL14ipa1* allele into Xiushui 11 (XS11) results in approximately an increase of 11% in grain yield [85]. Additional details on achievements of rice functional genomics have been well described in a recent review [86].

## Future perspectives

As the global human population is projected to reach 9 billion by 2050, rice researchers and breeders, together with those working on the other two major cereal crops – wheat and corn – are pressured to forge ahead to make decisive contributions to the prevention of potential food crises along the way. To fulfill such a challenging achievement, rice genomics and information integration must be conducted continuously, and an all-in effort from the rice research community would be needed to build the ICRG as a platform for the curation and annotation of sharable resources, which are not limited to data and knowledge but also experimental materials (Figure 1).

First, together with the introduction and application of the third-generation sequencers, we envisage that ICRG will contain more high-quality, gap-free genome sequences acquired systematically in the next decade or so from the existing germplasms, which may be expanded to other cereal crops and their wild counterparts. An international effort is in principle the best choice to unite rice scientists around the globe to build a platform upon which to collect data, to exchange information, and to share knowledge. As data accumulate, this platform must be organized to incorporate information from multiple omics levels, such as epigenomics, ribogenomics, proteomics, and metabolomics. Second, a significant effort must focus on gene-level genome annotations based on intensive comparative analyses among cultivars, wild counterparts, and elite hybrids. Most difficulties are expected to come from three basic components: defining all functional genes and their variants, annotating all TEs, and distinguishing orthologous and paralogous genes and their functional distinctions. Specialized databases have to be built and curated by dedicated scientists, in which genome polyploidy and chromosomal level regulatory principles and mechanisms are most likely involved. Third, ICRG dedicated to rice biotechnology must be built by rice genomicists and bioinformaticians for end users, such as rice biologists and crop breeders. Because the most likely tools for genetically modified crops now appear to be genome or gene editing in addition to the conventional tools of genetic engineering and hybridization [87], it is a necessity that genome assemblies be of ultimate quality and contiguity. Both are not effortless when working with the current state-of-art technological toolboxes. All possible future milestones are marked in the timeline of rice genomics (Figure 1A).

## Competing interests

The authors declare no competing interests.
